# 577. Conduction of a Burn Mass Casualty Incident Exercise at an Urban, Verified Burn Center

**DOI:** 10.1093/jbcr/irag033.340

**Published:** 2026-04-06

**Authors:** David G'sell, James E Johnson, Jeffrey E Carter, M Victoria P Miles, Jonathan E Schoen, Mario Rivera

**Affiliations:** University Medical Center, Louisiana State University Health Sciences Center, New Orleans, Metairie, LA; Wake Forest, Winston-Salem, North Carolina; University Medical Center Burn Center, Louisiana State University Health Sciences Center New Orleans, New Orleans, LA; University Medical Center Burn Center, Louisiana State University Health Sciences Center, Department of Surgery, New Orleans, New Orleans, LA; UCI Health Regional Burn Center, New Orleans, LA; The Burn Center at University Medical Center New Orleans, New Orleans, LA

## Abstract

**Introduction:**

Burn mass casualty incidents (BMCI) are indiscriminate and unpredictable, leading to significant logistical and clinical challenges for providers and facilities. In 2025, a verified trauma and burn center in the region was tasked with preparing for multiple National Special Security Events (NSSE), despite having experienced two BMCI involving more than 20 patients within the past four years. To enhance readiness, the burn center implemented a simulated BMCI using high-fidelity human patient simulators, moulaged burn survivors serving as standardized patient actors (SPA), and instructor-led scenarios. This report provides a descriptive account of the event to support other centers pursuing similar preparation efforts.

**Methods:**

The exercise, conducted in the first quarter of 2025, simulated the arrival of four burn patients within a 45-minute period, alongside a report of 12 total patients injured. Scheduled burn center staff were notified of the incident, divided into teams, and assigned roles. Model patients included two volunteer burn survivors and two healthcare simulation mannequins. Injuries ranged from 30%–36% TBSA deep partial-thickness and full-thickness burns to associated trauma such as fractures and inhalation injuries. Patients were triaged in the emergency department trauma bays and transferred accordingly. Scenarios were facilitated by senior interdisciplinary team members who provided structured feedback to participants, SPAs, and mannequins. Following the simulation, after-action reviews (AAR) and debriefing sessions were conducted.

**Results:**

The exercise identified critical aspects of burn care during a mass casualty scenario, with insights on communication and clinical management. Key lessons included the necessity of clearly defined duties and responsibilities, the value of closed-loop communication across multiple channels, and the importance of strategic oversight by clinical leaders such as the Burn Attending and Burn Charge Nurse. The exercise also demonstrated the need for adaptability, as unforeseen challenges may arise, requiring flexible responses and preparation strategies such as maintaining an emergency department burn disaster cart for rapid deployment and restocking.

**Conclusions:**

The simulation effectively evaluated burn center preparedness for a BMCI. Findings highlighted the importance of organized leadership, structured communication, and clinical adaptability in managing complex mass casualty events. The inclusion of burn survivors as standardized patient actors provided unmatched realism and feedback. These outcomes will guide future training initiatives and protocol development to optimize patient care during actual burn mass casualty events.

**Applicability of Research to Practice:**

The findings offer a framework for other institutions to design and implement local simulation and BMCI training programs.

**Funding for the study:**

This study was supported by the Spirit of Charity Foundation Burn Fund.

